# Association Between Myasthenia Gravis and Memory: A Systematic Review and Meta-Analysis

**DOI:** 10.3389/fneur.2021.680141

**Published:** 2021-11-19

**Authors:** Xiaoling Zhou, Yifei Zhou, Jianian Hua, Qun Xue

**Affiliations:** ^1^Department of Neurology, The First Affiliated Hospital of Soochow University, Suzhou, China; ^2^Medical College of Soochow University, Suzhou, China; ^3^Department of Gastroenterology, Jinling Hospital, Medical School of Nanjing University, Nanjing, China

**Keywords:** myasthenia gravis, memory, immediate recall memory, delayed recall memory, neuropsychology, cognition, meta-analysis

## Abstract

**Objective:** The studies have produced contradictory results regarding the association between myasthenia gravis (MG) and cognitive function, especially for the cognitive domains of memory. This meta-analysis was dedicated to exploring the association between MG and memory, which was represented by the immediate recall and delayed recall.

**Methods:** Using the random effects models, this study analyzed memory in MG based on data from the studies retrieved from four electronic databases from inception to February 2021. Disease severity was graded according to the Myasthenia Gravis Foundation of America (MGFA) classification. We defined ocular myasthenia gravis (OMG) (MGFA Grade I) as Class I, mild, and moderate generalized myasthenia gravis (GMG) (MGFA Grade IIa, IIb, IIIa, and IIIb) as Class II.

**Results:** In total, eight studies of 274 patients and 211 healthy controls were included. The significant associations were found between MG and memory. Compared with the healthy control group, the patients with MG performed significantly worse in the terms of immediate recall [standardized mean difference (SMD) = −0.65, 95% *CI* = −0.97 to −0.33, *P* < 0.001, *I*^2^ = 64.1%] and delayed recall (SMD = −0.49, 95% *CI* = −0.88 to −0.1, *P* < 0.05, *I*^2^ = 76.3%). Compared with the patients with Class I MG, those with Class II MG did not have significantly different scores in immediate recall (SMD = −0.07, 95% CI = −0.35 to 0.21, *P* = 0.614, *I*^2^ = 0%) and delayed recall (SMD = 0.63, 95% *CI* = −0.29 to 1.55, *P* = 0.178, *I*^2^ = 87.9%).

**Conclusion:** The patients with MG showed lower memory performance, such as both immediate and delayed recall ability. There was no association between the severity of MG and memory. Future studies should address whether these associations are casual and modifiable.

## Introduction

Myasthenia gravis (MG) is an autoimmune disease in which the antibodies bind to the acetylcholine receptors at the neuromuscular junction. Its major clinical manifestation is increased fatiguability of the voluntary muscles (1). Traditionally, it is defined as a disease with purely motor manifestations, but a recent study found that the patients with MG have non-motor symptoms, such as headache, sleep disorder, and cognitive and psychosocial issues (2). Nearly 60% of the individuals with MG complain of memory difficulties (3). Sabre et al. (4) established the MuSK^+^ PTMG (passive transfer myasthenia gravis) mouse model and discovered that recognition memory in the perirhinal cortex could be affected in MuSK^+^ MG mice. Mao et al. (5) reported that the patients with MG seem to perform more poorly than healthy controls in the terms of verbal learning and memory.

Nevertheless, some neuropsychological studies have denied that the patients with MG have memory impairment. Feldmann et al. (6) administered a battery of cognitive measures to 23 individuals with MG and 23 healthy control subjects. The results revealed that the significant group differences were not evident in terms of memory. Marra et al. (7) carried out a comprehensive neuropsychological test battery on 100 patients with MG and 31 matched control subjects, and the results showed that there were no differences in terms of memory between the patients and controls. The psychosocial and cognitive aspects represent an emerging field of research and clinical interest. Exploring the relationship between memory and MG may have broad prospects for the comprehensive treatment of MG in the future.

To date, the studies have produced contradictory results regarding the association between MG and cognitive function, especially for the cognitive domains of memory. Therefore, we conducted this systemic review and meta-analysis to (1) explore the association between MG and memory (immediate recall memory and delayed recall memory) and (2) assess whether memory (immediate recall memory and delayed recall memory) is related to the severity of MG.

## Methods

We reported our meta-analysis according to the Preferred Reporting Items for Systematic Reviews and Meta-Analysis (PRISMA) guidelines (8).

### Search Strategy

We conducted a systematic computerized search of the literature to identify relevant studies in Medline, Embase, Web of Science, and PsycINFO from inception to February 2021, combined with a manual search of reference lists from the identified articles. The search terms for each database are included in Supplementary Appendix 1. The search strategy combined the terms characterizing MG as the exposure variable and memory as the outcome variable. The literature searches under consideration were not restricted to the English-language articles. In addition, the journals, the conference abstracts, and the relevant references of the previous systemic reviews and included studies were searched.

### Selection Criteria

Based on the population, intervention/exposure, control, comparison, outcome, and study (PICOS) criteria, we defined the following selection criteria.

- P: The study population was the patients with MG without myasthenic crisis, a history of alcohol or drug abuse, severe respiratory or cardiovascular diseases, major psychiatric illness, or additional neurological illness, other disorders that may cause cognitive decline.
- I: A battery of neuropsychological tests was performed.
- C: The healthy control individuals selected by the original authors, matched for the age and education of the patients, without a history of alcohol or drug abuse, without severe respiratory or cardiovascular diseases, without major psychiatric illness or additional neurological illness, without other disorders that may cause cognitive decline.
- O: Memory, which was represented by the immediate recall and delayed recall.
- S: The cross-sectional studies.

**Figure 1:**
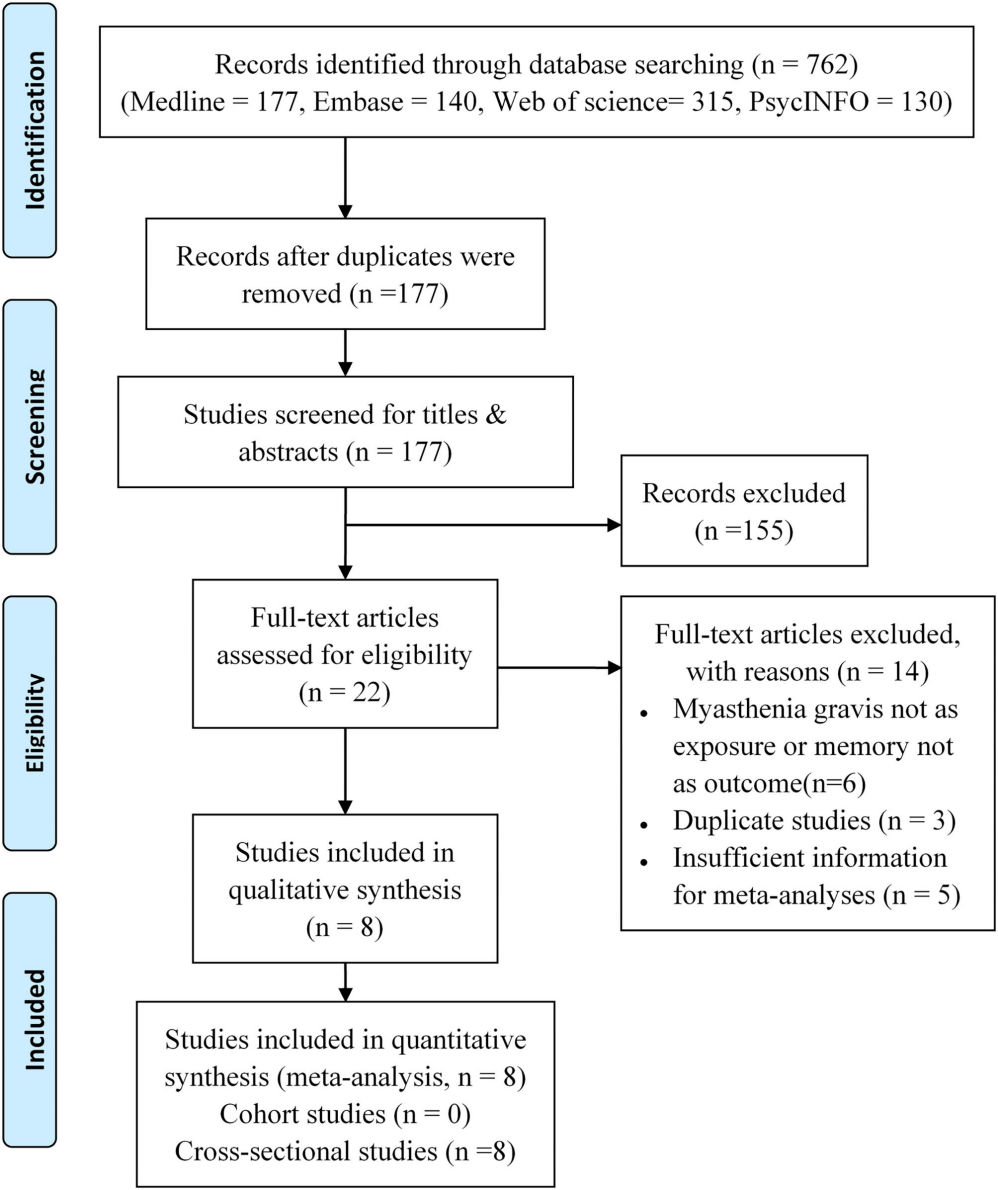
Flowchart for the included studies.

The inclusion of the studies was conducted in two phases: (1) screening of the title and abstract and (2) screening of the full text (Figure 1).

### Data Extraction

Two authors (XLZ and JNH) independently extracted all the useful data of each study involved in this meta-analysis. The conflicts were discussed with a third investigator (QX). The following information was extracted from each included study: the first author and publication year, study location (country and continent), study design, outcome measurements (memory measurement), demographic characteristics of MG group and control group (sample size, age, sex, and education), the pathophysiological and psychological characteristics of MG patients (disease duration, antibody type, treatment, and mood measurement), and main results. We attempted to contact the corresponding authors of the original articles to collect detailed information if the data were deficient or missing.

### Exposure and Outcome Measures

The diagnostic criteria of MG were the same in the included studies (1). Disease severity was graded according to the Myasthenia Gravis Foundation of America (MGFA) classification, and the patients were classified as Grade I, Grade II (IIa and IIb), Grade III (IIIa and IIIb), Grade IV, and Grade V (15). We defined ocular myasthenia gravis (OMG) (MGFA Grade I) as Class I, mild and moderate generalized myasthenia gravis (GMG) (MGFA Grade IIa, IIb, IIIa, and IIIb) as Class II. We extracted the data of Class I and Class II to explore the relationship between the severity of MG and memory.

Regarding the measurement of memory, all the studies used the subjective measurements, such as questionnaires. Since the definition of memory varies among the studies (16), two categories of memory (immediate recall memory and delayed recall memory) were extracted in our meta-analysis. Five articles used the auditory verbal learning test (AVLT) (17) and California verbal learning test (CVLT) (18) to assess memory. One study adopted immediate and delayed logical memory (19) to test memory. In brief, the subjects were told a detailed short story and were asked to recall the story immediately and again 30 min later. One study used the Randt memory test (RMT) (20) to evaluate memory. The RMT consists of seven different subtests (general information, five items, digit span, paired words, short story, picture recognition, and incidental learning) and three total scores (acquisition, recall, and memory index) are derived from them. Test administration includes a phase of acquisition from the seven different subtests. The recall phase consisted of recall results after interference and the results of delayed recall after 24 h. The remaining one study measured memory using the Wechsler memory scale-III (21).

### Mood Measurement

Considering the possible effects of moods, such as anxiety and depression on cognition, five studies used the clinical scales, such as the Self-rating Depression Scale (22), Profile of mood states, and Beck depression inventory (BDI) (23) to assess depression. Beck depression inventory was administered to assess the depression in our meta-analysis.

### Quality Appraisal

Study quality was assessed according to the nine-point Newcastle-Ottawa scale (NOS) (24). For the comparability category and exposure category, all the studies had a similar moderate quality. The main difference between the studies depends on the representativeness of the cases (Supplementary Table 1).

### Statistical Analysis

We searched the cohort studies and cross-sectional studies investigating the association between MG and cognitive function, especially for the cognitive domains of memory, but there were almost no cohort studies in the databases we searched. Therefore, we included eight cross-sectional studies that met the selection criteria.

In the analysis of the cross-sectional studies, we used the random effect models to estimate the pooled standardized mean differences (SMDs) and 95% *CI*s. Heterogeneity between the included studies was assessed by using the Cochran's Q test (significance level at *P* < 0.1) and *I*^2^ statistics (significance level at *I*^2^ > 50%) (25).

To explore the potential sources of heterogeneity, we divided the included studies into five groups based on geographic region, research time, memory test method, sample size, and study quality. This meta-analysis was performed using Stata 15.1 (StataCorp LLC, TX, USA).

A sensitivity analysis was conducted by excluding each study one by one to assess the robustness of the pooled results. We adopted Begg's test, Egger's test, and Egger's funnel plot to examine publication bias (26, 27).

## Results

### Study Characteristics and Study Quality

By searching the Medline, Embase, Web of Science, and PsycINFO, we identified 762 records in total. There were 177 records left for screening after removing the duplicated studies. On the basis of titles or abstracts, 22 studies were read by full text, and eight studies were included in the final analysis (6, 7, 9–14). Figure 1 shows the complete procedure for study selection and exclusion.

We incorporated eight cross-sectional studies that investigated the relationship between MG and memory. The number of patients ranged from 16 to 83 in all the eligible studies. All the studies used healthy people as a control, and the two groups had similar ages and education levels. The characteristics of the studies included in the meta-analysis are presented in Table 1. The results of the study quality assessment are shown in Supplementary Table 1. The NOS scores of all the eligible studies in our meta-analysis were higher than six points, indicating good study quality. The pathophysiological and psychological characteristics of the patients with MG are summarized in Table 2.

**Table 1:** The characteristics of the studies included in the meta-analysis.

**Author (Ref.)**	**Year**	**Country continent**	**Study design**	**Memory test**	**MG patients**				**Healthy controls**			
					**No**.	**% male**	**Age**	**Education**	**No**.	**% male**	**Age**	**Education**
Wang et al. (9)	2020	China Asia	C-S	CVLT	83	54	55.5 (12.2)	12 (4.5)	39	54.2	51.5 (12.5)	12 (4.4)
Eizaguirre et al. (10)	2017	Argentina America	C-S	Others	24	NR	43.9 (14.8)	10.9 (3.3)	24	NR	44.5 (15.4)	11.5 (3.3)
Marra et al. (7)	2009	Italy Europe	C-S	AVLT	40	58	71.8 (6.1)	8.6 (5.1)	31	48.4	72.8 (7.2)	9.1 (4.9)
Sitek et al. (11)	2009	USA America	C-S	AVLT	33	NR	47 (12)	12 (3)	30	NR	49 (12)	13 (3)
Feldmann et al. (6)	2005	Germany Europe	C-S	AVLT	23	52	46.7 (18.4)	NR	23	43.5	40.5 (13.4)	NR
Paul et al. (12)	2000	USA America	C-S	CVLT	28	NR	54.7 (13.4)	15.4 (2.7)	18	NR	51.2 (15.4)	16.4 (2.6)
Bartel et al. (13)	1995	Pretoria South Africa	C-S	Others	16	31	55 (NR)	8.2 (NR)	16	NR	NR	NR
Iwasaki et al. (14)	1990	Japan Asia	C-S	Others	27	30	41.56 (13.61)	12.4 (1.9)	27	NR	42.2 (13)	12.4 (1.7)

*C-S, cross-sectional study; CVLT, California verbal learning test; AVLT, auditory verbal learning test; NR, not recorded*.

**Table 2:** The pathophysiological and psychological characteristics of the patients with myasthenia gravis (MG).

**Characteristics of the subject population**	**Iwasaki (14)**	**Bartel (13)**	**Paul (12)**	**Feldmann (6)**	**Sitek (11)**	**Marra (7)**	**Eizaguirre (10)**	**Wang (9)**
No. of Patients	27	16	28	23	33	40	24	83
Disease Duration (years)	4.5	7.7	7.74 (6.71)	8.3	8 (7)	13.4 (21.8)	9.1 (8.5)	12 (3.4)
Antibody								
Anti-AchR-positive, *n* (%)	NR	NR	NR	17 (74)	NR	35 (87.5)	24 (100)	NR
Anti-AchR-negative, *n* (%)	NR	NR	NR	6 (26)	NR	5 (12.5)	0 (0)	NR
Anti-MuSK-positive, *n* (%)	NR	NR	NR	NR	NR	NR	NR	NR
Treatment								
ACHEI, *n* (%)	27 (100)	12	21 (75)	NR	19 (58)	37 (93)	NR	27 (33)
PRED, *n* (%)	0 (0)	12	13 (46)	NR	11 (33)	31 (78)	NR	32 (39)
IMMU, *n* (%)	0 (0)	4	11 (39)	NR	2 (6)	NR	NR	13 (16)
Mood Measurement								
Anxiety	NR	NR	NR	NR	NR	NR	NR	NR
Depression	SDS	POMS	NR	NR	BDI	NR	BDI	BDI

*OMG, ocular myasthenia gravis; GMG, generalized myasthenia gravis; ACHEI, acetylcholinesterase inhibitor; PRED, prednisone; IMMU, immunosuppressant; NR, not recorded; SDS, self-rating depression scale; POMS, profile of mood states; BDI, beck depression inventory*.

### Primary Analysis

#### The Association Between MG and Memory

**Figure 2:**
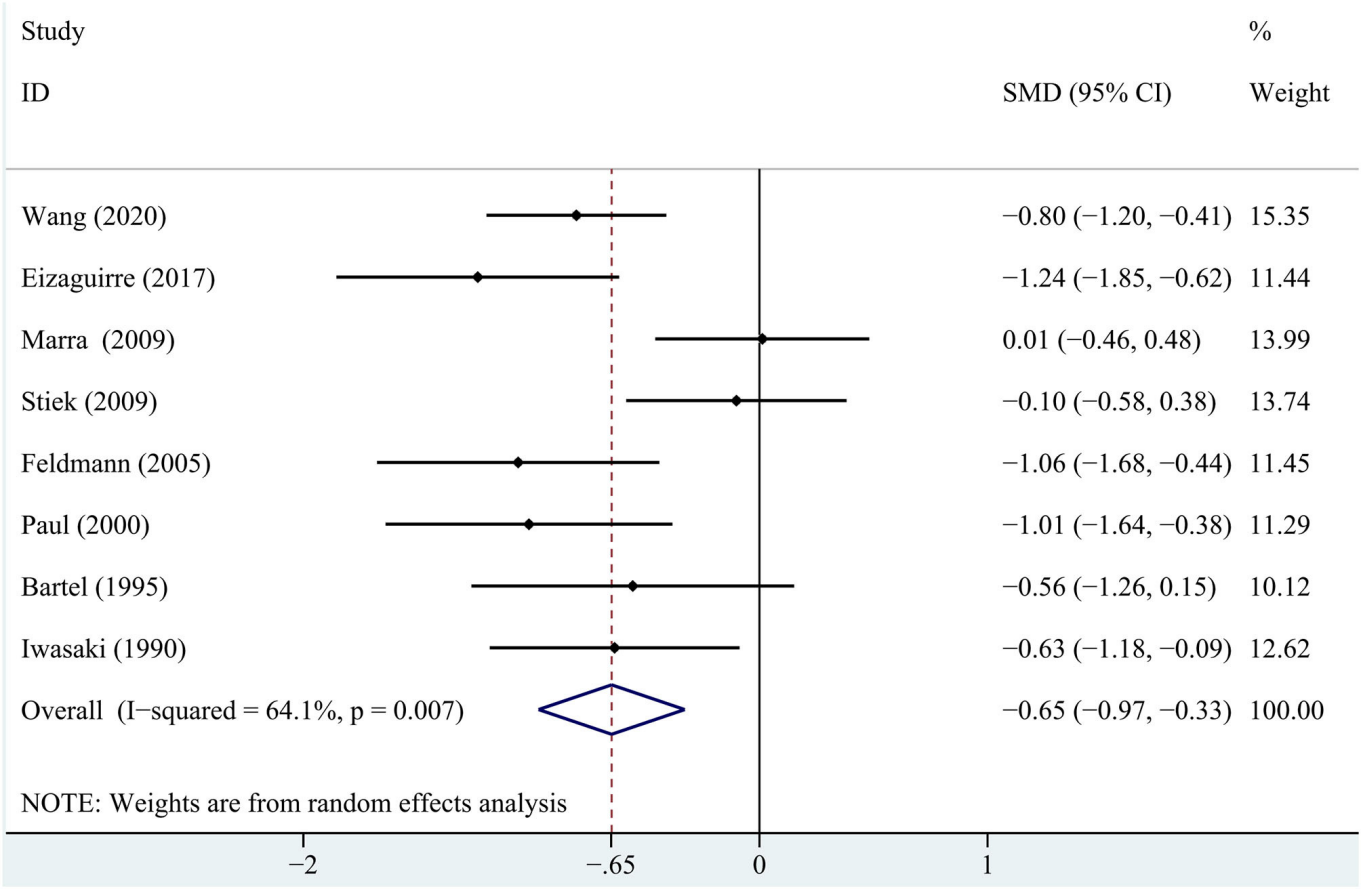
A forest plot of the association between myasthenia gravis (MG) and immediate recall memory.

In terms of immediate recall memory, the patients with MG performed significantly worse immediate recall score than the healthy control group (SMD = −0.65, 95% *CI*: −0.97, −0.33, *P* < 0.001, *I*^2^ = 64.1%; Figure 2).

**Figure 3:**
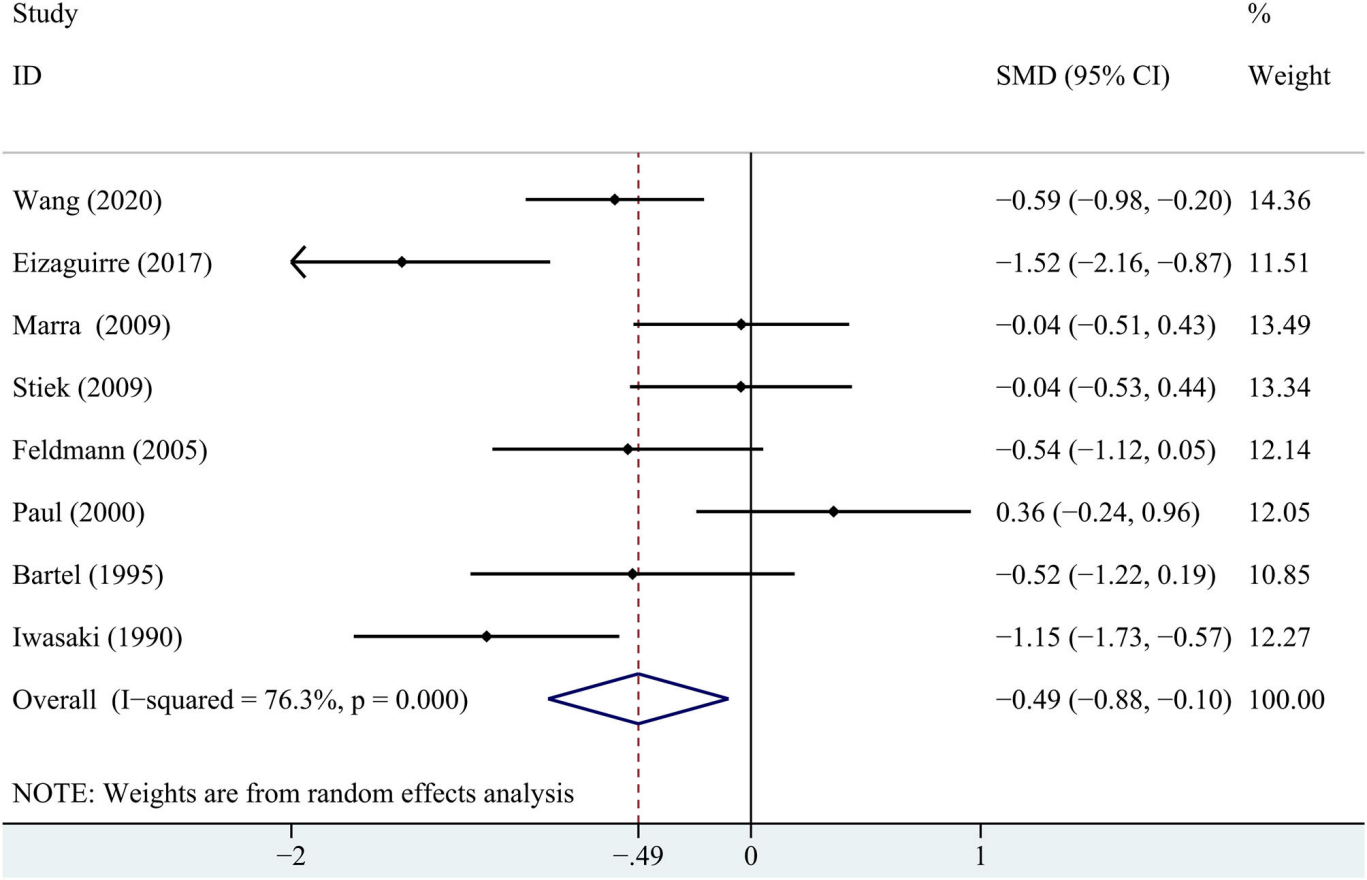
A forest plot of the association between MG and delayed recall memory.

Regarding the delayed recall memory, the results suggested that the patients with MG had significantly lower delayed recall score compared with the healthy controls (SMD = −0.49, 95% *CI*: −0.88 −0.10, *P* < 0.05, *I*^2^ = 76.3%; Figure 3). The effect size differences for memory, together with their *CI*s, significance tests, and homogeneity statistics, are presented in Table 3.

**Table 3:** Effect size statistics.

**Memory**	**Studies**	**No**.	**Effect size**	**95% CI**		***Z***	***P***	**Homogeneity statistics**	
			**(Cohen *d*)**	**LL**	**UL**			***I*** **^2^ (%)**	***P***
IRM	8	274	−0.65	−0.65	−0.97	3.97	<0.001	64.1	0.007
DRM	8	211	−0.49	−0.49	−0.88	2.45	0.014	76.3	<0.001

*IRM, immediate recall memory; DRM, delayed recall memory; LL, lower limit; UL, upper limit; CI, confidence interval*.

#### The Relationship Between the Severity of MG and Memory

**Figure 4:**
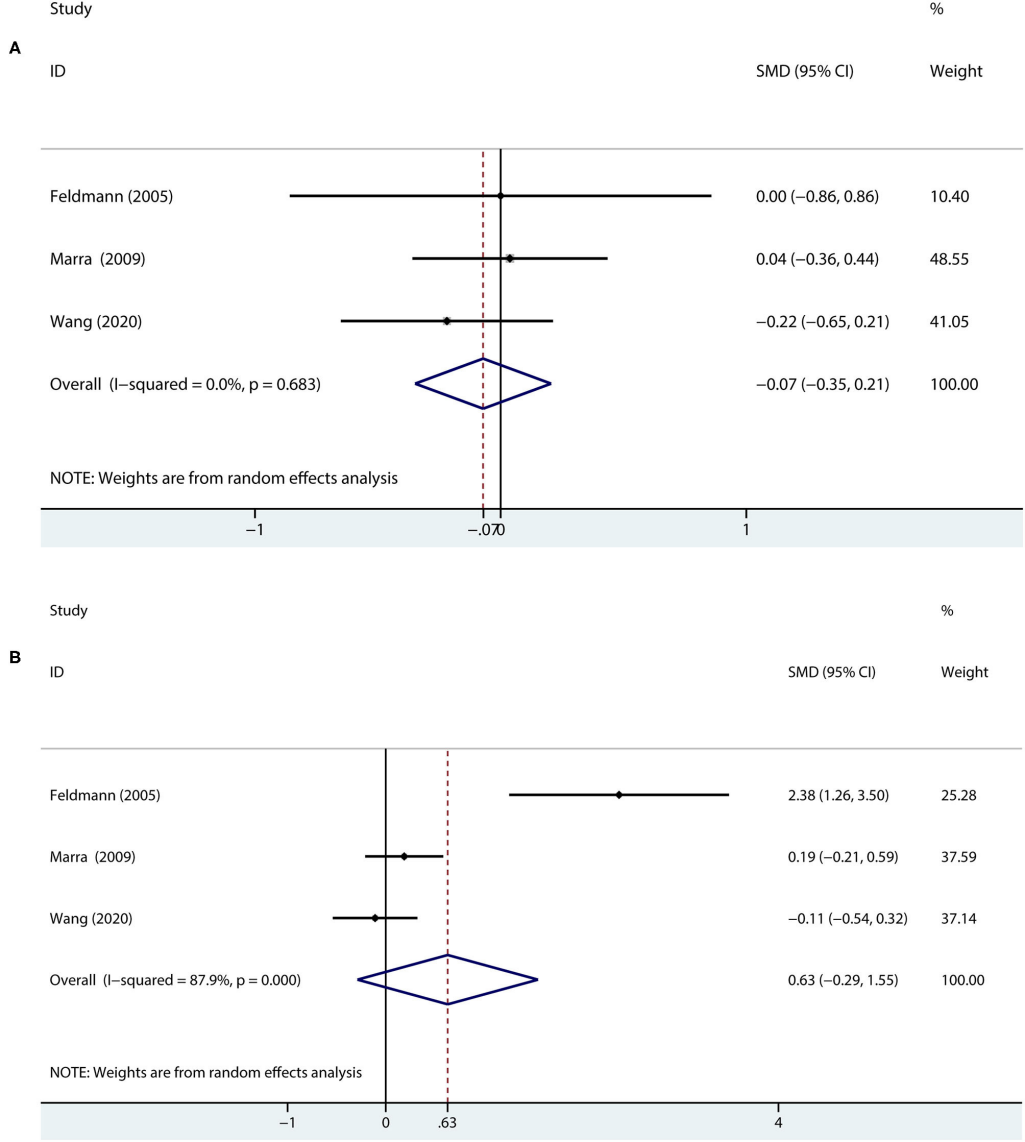
A forest plot of the association between the severity of MG and memory. **(A)** In terms of immediate recall memory, the patients with Class II MG were compared with the patients with Class I MG. **(B)** In terms of delayed recall memory, the patients with Class II MG were compared with the patients with Class I MG.

In terms of immediate recall memory, compared with the patients with Class I MG, those with Class II MG did not have significantly different scores (SMD = −0.07, 95% *CI* = −0.35 to 0.21, *P* = 0.614, *I*^2^ = 0.0%; Figure 4A) using a random effects model. The results were the same when using a fixed-effects model (data not shown).

Regarding delayed recall memory, the results showed that Class I and Class II MG patients were not statistically significant (SMD = 0.63, 95% *CI* = −0.29 to 1.55, *P* = 0.178, *I*^2^ = 87.9%; Figure 4B) using a random effects model.

Therefore, the severity of MG may not be associated with memory.

#### The Influence of Depression on the Patients With MG

**Figure 5:**
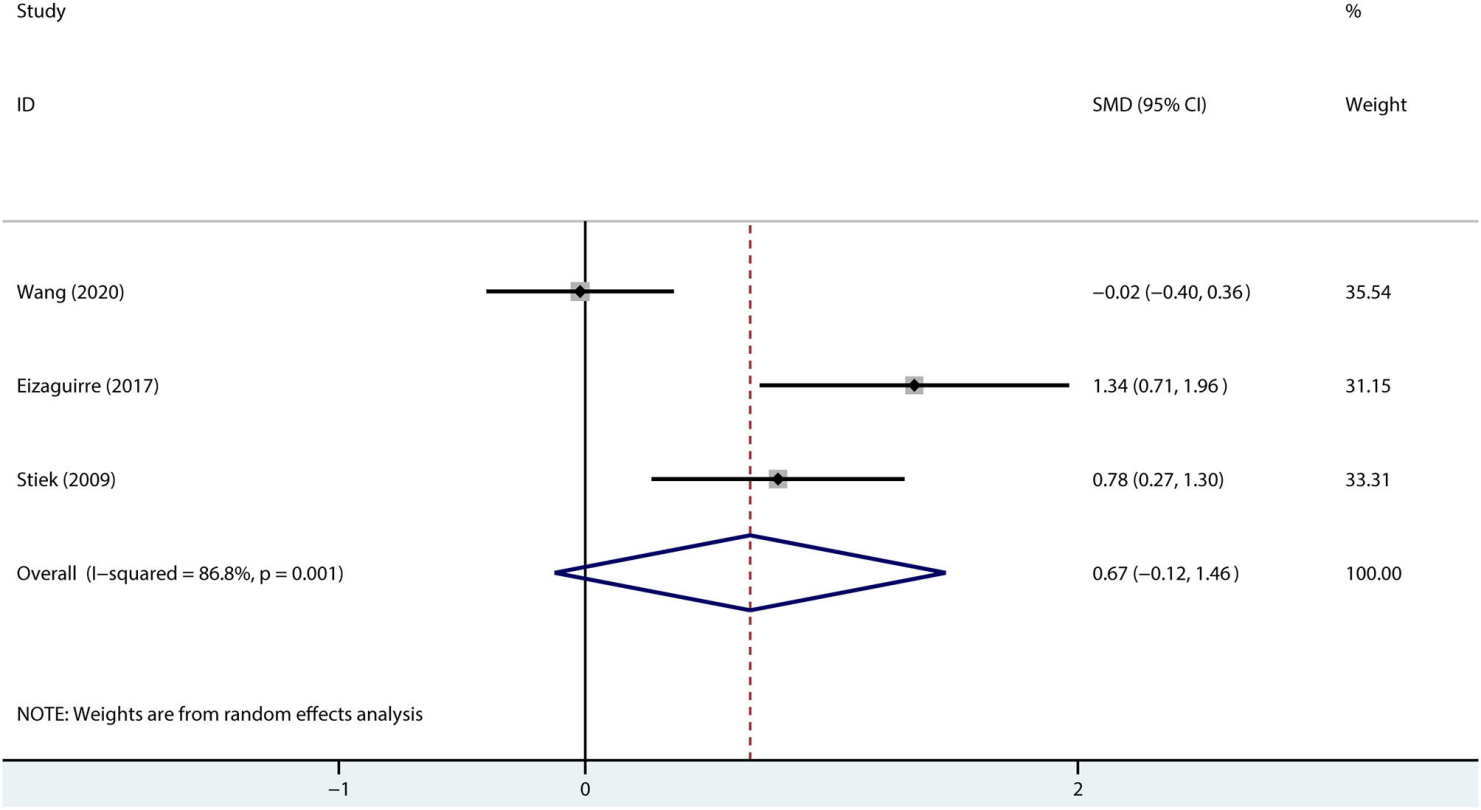
A forest plot of the association between MG and depression.

Given the possible influence of mood on cognition, we combined the depression scores to explore the association between the patients with MG and depression. The results suggested that compared with healthy controls, the patients with MG may not be accompanied by depression (SMD = 0.67, 95% *CI* = −0.12 to 1.46, *P* =0.098, *I*^2^ = 86.8%; Figure 5).

### Effect of Study Characters on the Heterogeneity

The results of the effect of study characters on heterogeneity are detailed in Table 4. We identified statistically significant effects in the groups we defined. Specifically, grouped by continent, the patients with MG in the Asia and America groups had significantly lower immediate recall score than healthy controls (Asia: SMD = −0.75, 95% *CI* = −1.06 to −0.43, *P* < 0.001, *I*^2^ = 0%; America: SMD = −0.76, 95% *CI* = −1.49 to −0.03, *P* = 0.041, *I*^2^ = 79.3%). When grouped by study time, the patients with MG in the studies prior to 2005 showed significantly lower immediate recall scores than healthy controls (SMD = −0.82, 95% *CI* = −1.12 to −0.51, *P* = 0.000, *I*^2^ = 0%). According to sample size grouping, immediate recall score of the patients with MG in the small sample size group (≤30) were significantly lower than those of healthy controls (SMD= −0.90, 95% *CI* = −1.18 to −0.62, *P* = 0.000, *I*^2^ = 0%). In accordance with the study quality grouping, the patients with MG in the low-quality group presented significantly worse immediate recall score than healthy controls (SMD = −0.87, 95% *CI* = −1.86, *P* = 0.000, *I*^2^ = 5%). Regarding delayed recall memory, the results showed that the patients with MG performed worse than the healthy control subjects in the Asia group (SMD = −0.83, 95% *CI* = −1.37 to −0.29, *P* = 0.003, *I*^2^ = 59.6%), and the low-quality group (SMD = −0.94, 95% *CI* = −1.41 to −0.46, *P* = 0.000, *I*^2^ = 55.9%). No statistically significant differences were seen between the groups stratified by research time, the memory test method, or sample size. Nevertheless, there was still unexplained heterogeneity (*I*^2^ > 50%) between the groups.

**Table 4:** Effect of study characters on the heterogeneity.

	**Immediate recall memory**				**Delayed recall memory**			
	**No. of studies (No. of patients)**	**SMD (95% CI)**	***I*** ^2^	***P***	**No. of studies (No. of patients)**	**SMD (95% CI)**	***I*** **^2^**	***P***
**Continent**								
Asia	2 (110)	−0.75 (−1.06, 0.43)	0.0	<0.001	2 (110)	−0.83 (−1.37, 0.29)	59.6	0.003
America	3 (85)	−0.76 (−1.49, 0.03)	79.3	0.041	3 (85)	−0.39 (−1.42, 0.64)	89.7	0.459
Europe	2 (63)	−0.50 (−1.55, 0.55)	86.3	0.347	2 (63)	−0.26 (−0.74, 0.22)	39.5	0.296
Africa	1 (16)	−0.65 (−0.97, 0.33)	–	0.124	1 (16)	−0.52 (−1.22, 0.19)	–	0.152
**Research Time**								
>2005	4 (180)	−0.51 (−1.05, 0.02)	79.8	0.061	4 (180)	−0.52 (−1.09, 0.05)	82	0.076
≤ 2005	4 (94)	−0.82 (−1.12, 0.51)	0.0	<0.001	4 (94)	−0.46 (−1.10, 0.17)	76.6	0.154
**Memory Test**								
CVLT	2 (111)	−0.86 (−1.19, 0.53)	0.0	<0.001	2 (111)	−0.14 (−1.08, 0.79)	85.4	0.761
AVLT	3 (96)	−0.35 (−0.96, 0.25)	75.3	0.254	3 (96)	−0.17 (−0.46, 0.13)	1.6	0.273
**Sample Size (MG)**								
>30	3 (156)	−0.31 (−0.84, 0.22)	76.1	0.249	3 (156)	−0.25 (−0.63, 0.13)	54.5	0.201
≤ 30	5 (118)	−0.90 (−1.18, 0.62)	0.0	<0.001	5 (118)	−0.67 (−1.31, 0.03)	81.1	0.309
**Study Quality**								
High	4 (184)	−0.46 (−0.94, 0.02)	74.7	0.061	4 (184)	−0.12 (−0.51, 0.28)	63.1	0.558
Low	4 (90)	−0.87 (−1.19, 0.56)	5.0	<0.001	4 (90)	−0.94 (−1.41, 0.46)	55.9	<0.001

*SMD, standardized mean differences; CI, confidence interval*.

### Sensitivity Analysis

None of the sensitivity analyses essentially changed the association between MG and memory, which was represented by the immediate recall and delayed recall (Supplementary Figure 1).

### Publication Bias

The specific effect sizes of the Begg's and Egger's tests were displayed in Supplementary Table 2. There was no significant publication bias. A visual inspection of the funnel plots also did not show apparent publication bias (Supplementary Figure 2).

## Discussion

Our article reviewed the association between MG and memory, and found the patients with MG had lower memory, such as both immediate and delayed recall, compared with the control groups. In addition, we found no relationship between the severity of MG and the degree of memory impairment.

In addition, we analyzed the included studies in the groups according to the study characteristics, such as geographic region, research time, memory test method, sample size, and study quality. In terms of immediate recall memory, we found statistical significance in the groups we defined. However, in terms of delayed recall memory, there was no significant difference between the groups stratified by research time, the memory test method, and sample size (Table 4). The age and gender differences were also considered. However, the MG and control groups of all the included studies were matched for age and education, and most for gender. We could not perform the age and gender subgroup analyses due to the lack of adequate relevant data.

Most of the patients with MG take pyridostigmine, prednisone, or immunosuppressants, such as azathioprine and tacrolimus (28). Pyridostigmine is hydrophilic and therefore does not easily cross the blood-brain barrier. Azathioprine has no known effects on the central nervous system (CNS) because it is a non-steroidal immunosuppressant. A study found that localized delivery of tacrolimus can repair the damaged CNS (29). Therefore, these drugs are less likely to interfere with cognition. However, prednisone does have an effect on CNS function. The use of glucocorticosteroids can alter cognition and mood (30, 31). We took the effects of hormones on cognition into account but did not perform the subgroup, correlation, and regression analyses of hormones because of the lack of adequate relevant data.

The pathophysiological and psychological characteristics may have important effects on the cognition of the patients with MG, such as the differences in antibody type, disease duration, treatment, anxiety, and depression. Three studies were pooled to assess the effect of depression on the patients with MG, and the results found that the patients with MG may not be accompanied by depression in our study. However, only one study reported memory scores in the anti-acetylcholine receptor antibodies positive group, and no studies reported the data about the memory scores in the anti-acetylcholine receptor antibodies negative group, anti-MuSK antibodies positive group. Because the included studies were cross-sectional studies, no studies reported the pre-treatment vs. post-treatment memory scores of the patients. Due to the lack of adequate relevant data, we could not perform a meta-analysis or subgroup analysis of the relevant pathophysiology and psychophysiology characteristics of the patients with MG, such as differences in antibody type, duration of disease, treatment, and anxiety.

To date, four alternative explanations have emerged for the possible memory impairment in the patients with MG: (1) The hypothesis of central cholinergic system impairment in MG (32, 33). The nicotinic receptors are distributed in the subcortical and cortical regions of the brain, where they participate in the specific cognitive and non-cognitive processes. The peripheral acetylcholine receptor antibodies (AchR-Abs) have access to the central receptors, and this discovery has led to the assertion that central and peripheral function may be impaired in MG (34). (2) The nocturnal respiratory problems, such as hypoxia and hypercapnia due to respiratory muscle weakness could cause cognitive deficits in MG (35, 36). (3) As a result of increased physical and mental fatigue (37, 38). Cognitive fatigue was defined as decreased performance with sustained cognitive effort. (4) The possible influence of non-specific immunological processes.

The psychosocial and cognitive aspects of MG represent an emerging area of research and clinical interest, but large-scale data are scarce worldwide. The studies on MG and memory have produced contradictory results. Our meta-analysis concluded that the patients with MG may have memory difficulties based on the combination of data from the eight cross-sectional studies, which may provide other researchers with a comprehensive and improved perspective on the association between MG and cognitive function. Meanwhile, the results may offer the clinicians comprehensive treatment options for the patients with MG, such as compensatory cognitive training in multiple sclerosis (39) and neuropsychological therapy (40). Nevertheless, only three included studies explored the association between the severity of MG and memory, and the results showed that immediate and delayed recall memory may not be associated with the severity of MG. The patients with severe GMG (MGFA grade IV and V) were also considered in some of the included studies, but only a study (7) explored the relationship between severe GMG and memory, and the number of studies was too small to constitute a meta-analysis. The results describing the relationship between the severity of MG and memory need to be interpreted with caution.

Our research also had several limitations. First, sample size justification and the power analysis are the key elements of a study design (41); only eight studies were included in this meta-analysis, and the sample size was relatively small. Second, our studies mainly focused on the neuropsychological examinations, which are the subjective measurements, but lacked the imaging studies, such as functional MRI (fMRI) (42), which is a method of objective measurement. Third, our studies included in this meta-analysis were cross-sectional rather than longitudinal (43), which may influence the results. Fourth, the neuropsychological tests used to evaluate memory are incompletely identical between the countries and studies (44, 45). Five, we can only conclude the comparison between the patients with MG and healthy controls because most of the studies were not designed to compare the patients with different clinical characteristics. Details about the pathophysiological and psychological characteristics of the patients with MG were not mentioned in the included studies. It is worth noting the distinction between the patients with or without anxiety and depression, as well as the evaluation of the patients with MG according to antibody types (anti-AchR-positive, anti-AchR-negative, or anti-MuSK-positive, respectively) and disease duration (the recently diagnosed patients and patients with long duration, respectively). Finally, regarding the exclusion criteria of subjects, some earlier studies did not exclude the patients with MG with severe visual limitation and severe infection. For the treatment of MG, we have not ruled out a hormonal effect on cognition.

In future, on the one hand, the studies exploring the relationship between MG and cognitive function should include sufficient sample longitudinal follow-up studies to detect, at minimum, the effect sizes. On the other hand, the animal models should be established, and molecular biology and neuroimaging techniques should be used for deeper exploration. Ultimately, the relevant pathophysiological and psychological characteristics of the patients with MG should be considered and various interference factors should be controlled.

## Conclusion

By performing this meta-analysis, we finally concluded that the immediate and delayed recall memory of the patients with MG may decrease compared with control subjects. The memory may not be associated with the severity of MG. The results could be promising for the comprehensive clinical treatment of MG, especially cognitive fields, in the future.

## Data Availability Statement

The original contributions presented in the study are included in the article/Supplementary Material, further inquiries can be directed to the corresponding author/s.

## Author Contributions

XZ and YZ are the main study investigators and analyzed the data of the included studies. JH and QX designed this meta-analysis scheme. XZ and JH wrote the manuscript. QX polished the language and revised the article. All authors contributed to the manuscript equally and agreed to submit this meta-analysis.

## Funding

This study was supported by the Key R&D Program of Jiangsu Province (BE2019666) and the Jiangsu Natural Science Foundation (BK20211075).

## Conflict of Interest

The authors declare that the research was conducted in the absence of any commercial or financial relationships that could be construed as a potential conflict of interest.

## Publisher's Note

All claims expressed in this article are solely those of the authors and do not necessarily represent those of their affiliated organizations, or those of the publisher, the editors and the reviewers. Any product that may be evaluated in this article, or claim that may be made by its manufacturer, is not guaranteed or endorsed by the publisher.
