# Supplementary material for: Association Between Myasthenia Gravis and Memory: A Systematic Review and Meta-Analysis
Source: Front Neurol. 2021 Nov 19;12:680141. doi: 10.3389/fneur.2021.680141 (PMC8640249; doi:10.3389/fneur.2021.680141)
Supplement: Supplementary file 1 [file Data_Sheet_1.pdf]

Supplementary Materials

1 List of Captions

Captions	Contents
Table S1	Quality appraisal using the modified Ottawa-Newcastle Scale
Table S2	Publication bias
Figure S1	Sensitivity analyses
Figure S2	Funnel plots
Appendix S1	Search terms

2 Tables

2.1 Table S1. Quality assessment using the modified Ottawa-Newcastle Scale

Author, year	Design	Modiefied Ottawa-Newcastle Scale		
		Selection	Comparability	Exposure/outcome
Wang,2020	C-S	★★★	★★	★★
Eizaguirre,2017	C-S	★★	★★	★★
Marra,2009	C-S	★★★	★★	★★
Stiek,2009	C-S	★★★	★★	★★
Feldmann,2005	C-S	★★	★★	★★
Paul,2000	C-S	★★★	★★	★★
Bartel,1995	C-S	★★	★★	★★
Iwasaki,1900	C-S	★★	★★	★★

Abbreviations: C-S, cross-sectional study.

2.2 Table S2. Publication bias

	P for Egger test	P for Begg test
Immediate Recall Memory	0.364	0.386
Delayed Recall Memory	0.577	0.902

3 Figure legends

Figure S1. Sensitive analyses. Sensitivity analyses of association between myasthenia gravis and memory ability by omitting one study each time and rerunning the analyses. (A)immediate recall memory; (B) delayed recall memory.

Figure S2. Funnel plots. Funnel plots for the memory ability. (A) immediate recall memory; (B) delayed recall memory.

4 Appendix

4.1 Appendix S1. Search terms.

Search terms for MEDLINE through Pubmed (search data:Febryary 1, 2021)

("cognitive function"[All Fields] OR “cognition"[All Fields] OR “cognitive impairment"[All Fields] OR "cognitive disorder”[All Fields] OR "cognitive deficit” [All Fields] OR " neuropsychology "[All Fields] OR "memory"[All Fields] OR “memory ability”[All Fields]) AND (“myasthenia gravis”[All Fields] OR “MG” [All Fields] [All Fields])

Search terms for EMBASE through Ovid (search data:February 1, 2021)

1. cognitive function.ti,ab,kw
2. cognition.ti,ab,kw
3. cognitive impairment.ti,ab,kw
4. cognitive disorder.ti,ab,kw
5. cognitive deficit.ti,ab,kw
6. neuropsychology.ti,ab,kw
7. memory.ti,ab,kw
8. memory ability.ti,ab,kw
9. 1 OR 2 OR 3 OR 4 OR 5 OR 6 OR 7 OR 8
10. myasthenia gravis.ti,ab,kw
11. MG.ti,ab,kw
12. 11 OR 12
13. 9 AND 12

Search terms through Web of Science(search data:Febryary 1, 2021)

("cognitive function " OR “cognition " OR " cognitive impairment " OR " cognitive disorder " OR " cognitive deficit " OR "neuropsychology " OR " memory " OR " memory ability ") AND (“myasthenia gravis” OR “MG”)

Search terms for PsycINFO through Ovid (search data:Febryary 1, 2021)

1. cognitive function.tw
2. cognition.tw
3. cognitive impairment.tw
4. cognitive disorder.tw
5. cognitive deficit.tw
6. neuropsychology.tw
7. memory.tw
8. memory ability.tw
9. 1 OR 2 OR 3 OR 4 OR 5 OR 6 OR 7 OR 8
10. myasthenia gravis.tw
11. MG.tw
12. 10 OR 11
13. 9 AND 12
