# Supplementary figures and images for: Association Between Myasthenia Gravis and Memory: A Systematic Review and Meta-Analysis
Source: Front Neurol. 2021 Nov 19;12:680141. doi: 10.3389/fneur.2021.680141 (PMC8640249; doi:10.3389/fneur.2021.680141)

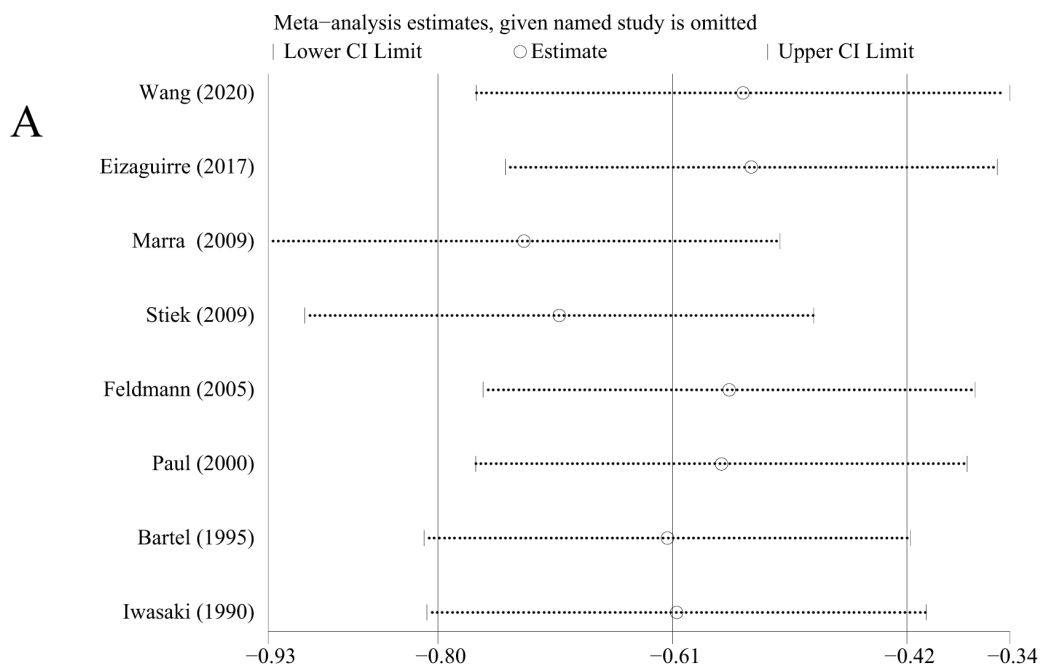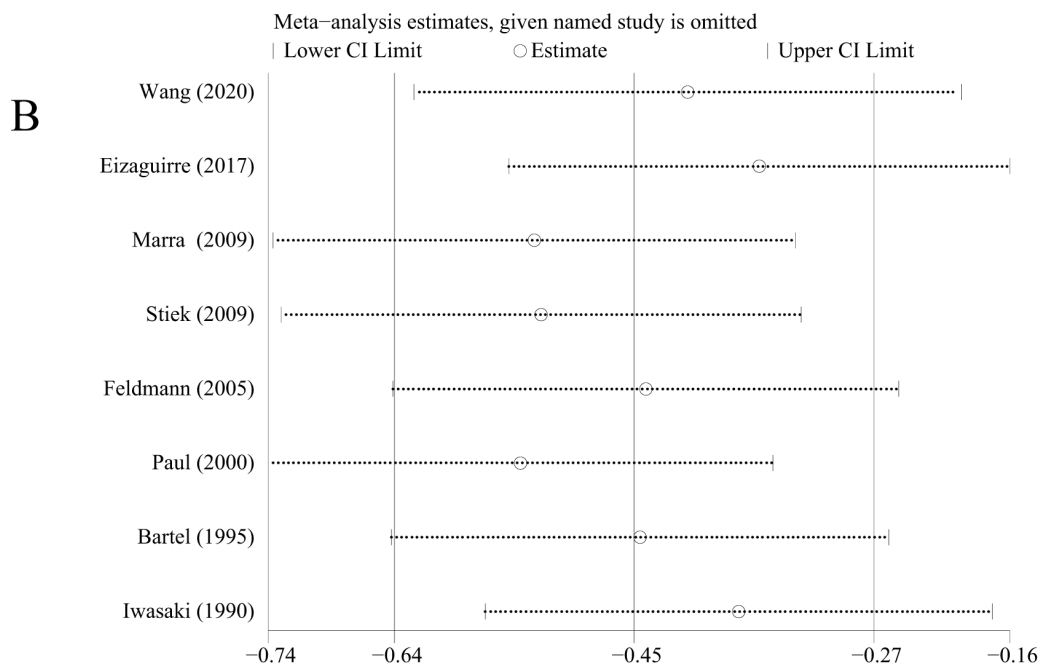

Supplement: Supplementary file 2 [file Image_1.pdf]

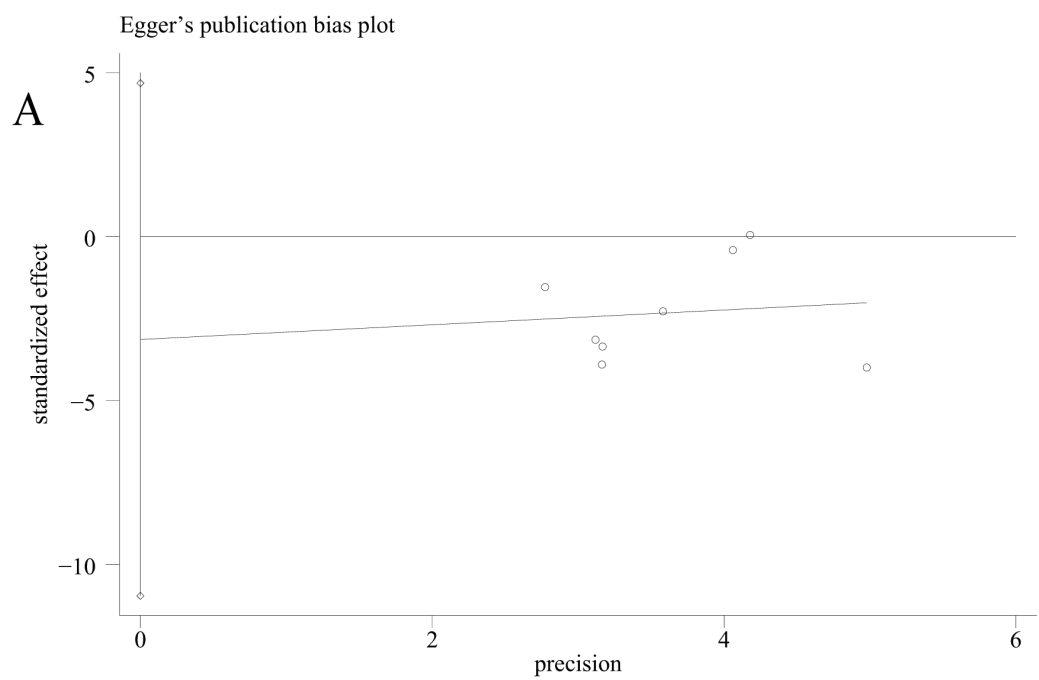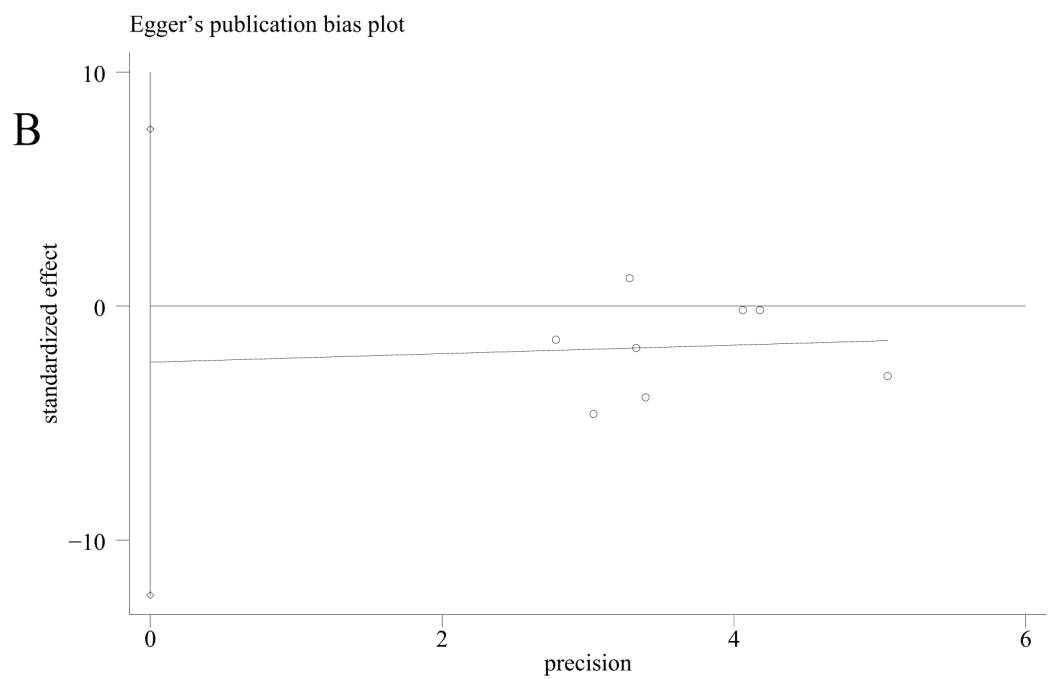

Supplement: Supplementary file 3 [file Image_2.pdf]
